# Neural correlates of user learning during long-term BCI training for the Cybathlon competition

**DOI:** 10.1186/s12984-022-01047-x

**Published:** 2022-07-05

**Authors:** Stefano Tortora, Gloria Beraldo, Francesco Bettella, Emanuela Formaggio, Maria Rubega, Alessandra Del Felice, Stefano Masiero, Ruggero Carli, Nicola Petrone, Emanuele Menegatti, Luca Tonin

**Affiliations:** 1 Department of Information Engineering, University of Padova, Padua, Italy; 2 Institute for Cognitive Science and Technology, National Research Council, Rome, Italy; 3 Department of Industrial Engineering, University of Padova, Padua, Italy; 4 Department of Neuroscience, Section of Rehabilitation, University of Padova, Padua, Italy; 5 Padova Neuroscience Center, University of Padova, Padua, Italy

**Keywords:** Mutual learning, User learning, Motor imagery, Brain-computer interface, Riemann geometry, Long-term evaluation, Cybathlon

## Abstract

**Background:**

Brain-computer interfaces (BCIs) are systems capable of translating human brain patterns, measured through electroencephalography (EEG), into commands for an external device. Despite the great advances in machine learning solutions to enhance the performance of BCI decoders, the translational impact of this technology remains elusive. The reliability of BCIs is often unsatisfactory for end-users, limiting their application outside a laboratory environment.

**Methods:**

We present the analysis on the data acquired from an end-user during the preparation for two Cybathlon competitions, where our pilot won the gold medal twice in a row. These data are of particular interest given the mutual learning approach adopted during the longitudinal training phase (8 months), the long training break in between the two events (1 year) and the demanding evaluation scenario. A multifaceted perspective on long-term user learning is proposed: we enriched the information gathered through conventional metrics (e.g., accuracy, application performances) by investigating novel neural correlates of learning in different neural domains.

**Results:**

First, we showed that by focusing the training on user learning, the pilot was capable of significantly improving his performance over time even with infrequent decoder re-calibrations. Second, we revealed that the analysis of the within-class modifications of the pilot’s neural patterns in the Riemannian domain is more effective in tracking the acquisition and the stabilization of BCI skills, especially after the 1-year break. These results further confirmed the key role of mutual learning in the acquisition of BCI skills, and particularly highlighted the importance of user learning as a key to enhance BCI reliability.

**Conclusion:**

We firmly believe that our work may open new perspectives and fuel discussions in the BCI field to shift the focus of future research: not only to the machine learning of the decoder, but also in investigating novel training procedures to boost the user learning and the stability of the BCI skills in the long-term. To this end, the analyses and the metrics proposed could be used to monitor the user learning during training and provide a marker guiding the decoder re-calibration to maximize the mutual adaptation of the user to the BCI system.

**Supplementary Information:**

The online version contains supplementary material available at 10.1186/s12984-022-01047-x.

## Background

A brain-computer interface (BCI) is, by definition, composed by two intelligent actors: the user and the neural decoder [1, 2]. On the one hand, the BCI user actively performs a given mental task by responding to an external stimulus or by voluntary self-modulating the brain signals. On the other hand, the decoder is responsible for classifying and interpreting the user’s neural signals in order to translate them into commands for an external device [1, 3].

Following this approach, several studies demonstrated the potentiality of BCI as an assistive technology to control a variety of brain-actuated prototypes (e.g., computer-based applications, telepresence robots, powered wheelchairs [4]) and, eventually, to restore the independence of those people suffering from severe motor disabilities [5, 6].

However, despite the great advances and after decades of research, the translational impact of BCI technology remains elusive. Indeed, in most cases BCI reliability is still unsatisfactory for end-users. In addition, current protocols to calibrate the BCI are long and tiring, while the robustness and the stability over time of the performances are often limited [5]. As a consequence, current evaluations of BCI systems mostly report on able-bodied users or on end-users during short-term and control study scenarios [7–11].

The last decades have seen a growing and wide-spread tendency of facing these challenges by focusing on the learning processes of the second actor involved in the BCI system, the neural decoder. This is probably due to the vast research in the domain of stimulus driven BCIs (e.g., based on P300 evoked potentials or (SSVEPs)) where the space for the user to learn how to modulate his/her neural rhythms is limited. Several machine learning solutions have been proposed to handle the variability of the BCI, to stabilize the performances, to increase the number of possible commands and even to achieve out-of-the-box BCI systems that do not need either training nor calibration to decode user’s intentions [12–22]. However, to date, these approaches were not efficient in managing the current translational limitations of BCI systems.

On the contrary, recent evidence identifies a more holistic approach as a possible solution for improving the BCI reliability. This approach is based on the concept of mutual learning in which both BCI actors (user and decoder) need to concurrently adapt and learn from each other in order to achieve optimal BCI control [1, 2]. The hypothesis that BCI is a skill to be learned through mutual learning has been already introduced in the early years of research, and especially in the invasive BCI community [20, 21, 23–30]. However, recent studies highlighted the importance of user/decoder training and mutual learning also in noninvasive BCIs (e.g., based on electroencephalography (EEG)) and especially in the case of interfaces based on the voluntary self-regulation of brain rhythms (e.g.,BCIs driven by motor imagination) [31–38]. These studies demonstrated that robust BCI control may be achieved by systematically targeting improvements of both user learning and machine learning during the training period. In particular, works from [34–38] report on the experience in the BCI Race Discipline of the Cybathlon events (i.e., 2016 Cybathlon, 2019 Cybathlon BCI Series in Graz and the 2020 Cybathlon Global edition). While in [37, 38] the importance of the training and the user’s engagement during closed-loop BCI operations has been clearly highlighted, in [35, 36] the authors described the machine learning approaches for unsupervised adaptive re-calibration exploited by the teams to reduce intra- and inter-session variability of user’s brain signals. Perdikis et al. [34] mostly focused on the user learning evolution by reporting the longitudinal BCI training of two tetraplegic users for the first 2016 Cybathlon event. Results showed the clear correlation between the increasing acquisition of BCI skill (user learning) through mutual learning and the application performances that ended up with the discipline record and the gold medal victory during the event.

In this paper, we present the analysis on the data acquired during the preparation for the 2019 Cybathlon BCI Series in Graz and the 2020 Cybathlon Global Edition where our pilot won the gold medal—twice in a row– as a member of the WHI Team. We consider the data of particular interest given the mutual learning approach adopted during the longitudinal training phase (8 months), the long training break in between the two events (1 year) and the demanding evaluation scenario represented by the Cybathlon competition. As in [34], results have strengthened the hypothesis that mutual learning is the key for triggering user learning, especially in the first months of training. Moreover, in this study, we aimed at exploiting the longitudinal training protocol to further and deeply investigate the neural correlates of user learning by proposing a new metric that appears to be directly related to the acquisition of stable and robust BCI skills. We consider that this may represent the key not only for the success of our pilot during the Cybathlon events but also for improving the long-term reliability of BCI technology in real-world scenarios.

## Methods

### Pilot

Our pilot (male, 30 years old) is a paralympic swimming athlete. He was diagnosed at the age of two with Charcot Marie Tooth 2A. He had clinical bilateral severe weakness of distal upper and lower limbs (1/5 on Medical Research Council (MRC) Scale), and weakness of proximal muscles (3+/5 MRC on deltoide, biceps and triceps brachii and ileopsoas). Head and trunk control was maintained. His formal neurological examination fulfilled all the inclusion criteria for the Cybathlon BCI Discipline and he was eligible for participation to the competition.

He voluntary accepted to take part to both the competitions, the 2019 Cybathlon BCI Series and the 2020 Cybathlon Global Edition, and to the intensive training sessions over 2019-2020 years. He agreed to the recording and the analysis of his EEG activity for this purpose. Moreover, he has been actively involved in all the development phases of the BCI system used for the competitions.

### Cybathlon BCI Race

The Cybathlon BCI Race consists of a virtual game, called “BrainDriver”^1^, where the pilot was required to steer a brain-controlled vehicle through a race track. Each track is divided into 16 sections. The avatar could pass through four possible sections, equally distributed over the track: “right”, “headlight”, “left” and “noinput”. The first three are called “active sections” since in these sections the pilots are expected to send the appropriate in-game commands using the BCI system in order to make the avatar accelerate. The “noinput” sections correspond to areas where any command could be sent, otherwise the avatar decelerates.

During the race, pilots must watch the screen without receiving any additional stimulation and/or external feedback (e.g., about the decoder output). Any ocular control and any other muscular activity is not allowed. Any command affected by muscular artefacts is therefore automatically discarded (please refer to Section “EOG detection”).

### BCI implementation

#### SMR BCI

The BCI system adopted by the WHI team consists of a motor imagery (MI) BCI based on sensorimotor rhythms (SMRs) for class discrimination. In particular, the system was developed to discriminate between the imagination of the movement of both hands against both feet. The choice of the MI paradigm was taken after a screening session in which the separability of different mental tasks has been evaluated with our pilot. The selection of the both hands vs. both feet paradigm is also supported by the results obtained in previous BCI experiments [39, 40]. EEG was acquired with a lightweight, 16-channel g.USBamp amplifier (g.Tec medical engineering, Schiedelberg, Austria). The EEG signal was recorded at 512 Hz sampling rate, hardware filtered within 0.1 and 100 Hz, and notch-filtered at 50 Hz. For the MI BCI, 14 electrodes were placed over the sensorimotor cortex (Fz, FC3, FC1, FCz, FC2, FC4, C3, C1, Cz, C2, C4, CP1, CPz, CP2 of the 10-20 EEG system), with the ground location on the AFz electrode and referenced to the right earlobe. The BCI algorithms for data recording and processing, as well as for user training and game control were implemented in ROS-Neuro^2^, an open-source extension of the Robot Operating System (ROS) for brain-driven robotic applications [41–43]. The algorithms for the BCI system calibration were instead running offline in Matlab using a custom software library.

**Fig. 1:**
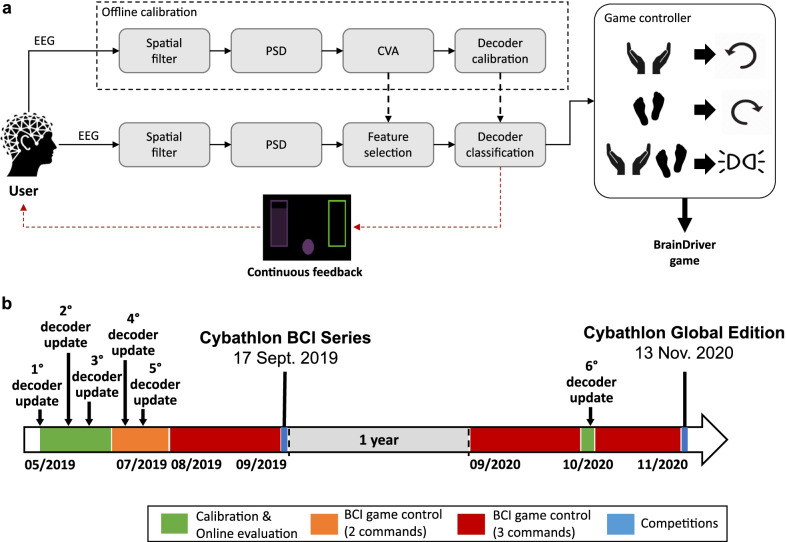
Overview of the BCI implementation and training protocol. **a** BCI pipeline to classify both hands and both feet motor imagery. First, the raw EEG signals were spatially filtered and their power spectral density (PSD) extracted. During the offline calibration, the most discriminative features were identified through canonical variate analysis (CVA) and used to calibrate the decoder to classify the two mental tasks. The BCI commands were then converted into the proper game commands to control the BrainDriver game. During the online evaluation only, continuous feedback about the decoder outputs were visually provided to the user to foster learning. **b** Timeline illustrating the pilot training protocol and the approximate day of decoder update from the first contact with the pilot to the day of the Cybathlon 2020 Global Edition. Between the end of the Cybathlon 2019 BCI Series (17/09/2019) to the following training session (15/09/2020), the pilot spent almost one year without using any BCI system

A schematic representation of the EEG processing and classification pipeline is shown in Fig. 1a. The EEG signal was first spatially filtered with a Laplacian mask using adjacent electrodes. The power spectral density (PSD) of the signal with 2 Hz resolution was computed via Welch’s algorithm using a 1 s-long Hamming window sliding every 62.5 ms. A semi-automatic feature selection was performed through canonical variate analysis (CVA) identifying the most discriminant spatiospectral features (i.e., channel-frequency pairs) according to the training data. The operators regularly checked for changes in the pilot’s feature maps, which were correlated with a decrease of performance, either quantitatively by looking to the classification accuracy or qualitatively by directly interrogating the pilot, in order to decide when an update of the system was required. Table 1 presents the spatiospectral features (bands and locations) selected in every system re-calibration. A Gaussian classifier was then trained with the selected features to model the probability distributions over the two MI tasks (i.e., both hands, both feet) and to compute the posterior class probabilities of each feature vector in real-time. The classifier’s parameters were initialized using self-organizing maps (SOM) clustering and trained with a gradient-descent supervised learning method. The samples whose maximum posterior probability was below a certain threshold were rejected (i.e., $th_{rej}=0.6$ selected by the operators’ experience), since they correspond to “uncertain” classifications. To increase the system reliability, the posterior probabilities were accumulated over time by means of an integration framework based on dynamical systems [39]. When the integrated probabilities of a class reach the value of 1, the corresponding BCI command is delivered. The integrator has been designed with a twofold purpose: to support the user in the delivery of the intended commands and, at the same time, to handle the possible erratic behavior of the BCI decoder output, thus preventing false positives. Upon delivery of a BCI command, the integrated probabilities are reset to the uniform distribution and a refractory period of 1 s is set between two consecutive commands.

**Table 1:** Features selected for decoder calibration

Date	Feature		Date	Feature	
	Location	Band [Hz]		Location	Band [Hz]
2019/05/02	FC2	20	2019/07/09	FC1	20
	FC2	22		FC1	22
	C4	20		FC2	20
	C4	22		FC2	22
2019/05/21	Fz	22		C4	18
	Cz	16		C4	20
	Cz	22		C4	22
	C4	20	2020/10/27	C3	22
	C4	22		C3	24
2019/06/27	FC2	20		C4	20
	FC2	22		C4	22
	C4	20		C4	24
	C4	22		CP2	22
2019/07/01	FC2	20		CP2	24
	FC2	22			
	C4	20			
	C4	22			

The table presents the date and all the spatio-spectral features selected for the BCI decoders trained throughout our pilot’s training periods. Each feature refers to a specific frequency band (2 Hz resolution) and EEG channel location according to the international 10-20 system

To translate the commands of our 2-class BCI into in-game commands and control the avatar in the four sections, we adopted the strategy which is described below. The “left” and “right” commands were controlled by both hands and both feet MI tasks, respectively. For the third “active section”, we decided to implement a sequential strategy: the “headlight” command was delivered when the pilot succeeded in sending two consecutive BCI commands of different types (i.e., both hands/both feet or both feet/both hands) within a configurable interval of time (e.g., 2 seconds). Thus, the pilot should first deliver to the game either a “right” or “left” command, through the corresponding MI task. If the following BCI command is generated within the interval of time and it is opposite to the previous command, then instead of sending a “right” or “left” command, the “headlight” command is delivered to the game. A schematic representation depicting the design of the implemented game control paradigm is shown in Additional file 1: Fig. S1. Although this approach requires the delivery of a wrong command before outputting the correct “headlight” command, similar approaches have been previously proposed to increase the degree of control over complex active devices using few mental tasks [44–46] and have shown successful performance in the previous Cybathlon 2016 edition [34]. The fourth game command, thus the one required to send no control commands to the game on the “noinput” sections, was implicitly obtained thanks to the integration framework of our BCI system. Indeed, when none of the integrated class probabilities of the two MI tasks reaches the value of 1, the subject is considered in an intentional non-control (INC) state [47], and no commands are sent to the game.

The implementation of our BCI system is available online^3^ and we refer the reader to [43] for a more detailed description of the software.

#### EOG detection

The implemented artifact control scheme targets the detection of electrooculography (EOG) and facial electromyogram (EMG) signals. The final goal is to freeze the BCI output and prevent any outgoing command towards the game while the presence of such artifacts is observed. To achieve a minimally obtrusive setup, only 2 electrodes (plus reference) are employed. Each electrode is placed above an eye (approximately Fp1 and Fp2 locations), while the reference is placed at the AFz location (10-20 system layout). EOG signals were acquired at 512 Hz in frames of 32 samples every 62.5 ms, synchronously with the EEG acquisition. Artifact detection is performed separately on each consecutive frame, resulting in very fluid and responsive detection of artifact onset and offset. For each frame, the two original channels are combined as follows to determine the horizontal and vertical EOG components:

1 $$\begin{gathered} HEOG = EOG_{{Fp1}} - EOG_{{Fp2}} \hfill \\ VEOG = \frac{{(EOG_{{Fp1}} + EOG_{{Fp2}} )}}{2} \hfill \\ \end{gathered}$$

Low-frequency content is extracted from the signals with a zero-lag second-order Butterworth filter in the 1–10 Hz frequency band [48, 49]. Signal mirroring is applied before filtering to avoid discontinuities at the edges of the time window. EOG amplitudes are then obtained as the absolute value of the horizontal and vertical components. If the amplitude of the horizontal and/or vertical EOG components exceeds a predefined threshold (i.e., 30 uV), the system prevents the BCI from sending commands for 2 seconds. The threshold of 30 uV has been selected for our pilot after empirical evaluation. During the races, the threshold value is kept constant.

### Training periods and strategy

In order to foster the mutual learning between the user and the machine, we adopted a three-stage training: *calibration*, *online evaluation*, *race control*. This strategy aimed at speeding up the creation of a reliable classifier with stable features, allowing the pilot to learn how to modulate his SMRs in the final application as soon as possible.

The pilot started the training in May 2019. Initially, an open-loop *calibration* acquisition of EEG data was performed. The protocol concerned the repetition of cue-guided trials of either both hands or both feet MI. After the appearance of a color-coded cue, informing the pilot on the task to be performed, each MI trial lasted from 4.5 s to 5.5 s. At this stage, a visual feedback, automatically moving towards the correct direction, was provided to the user. Each calibration run consisted of 15 trials for each MI task. Data from the first day (3 calibration runs) were used to calibrate the BCI classifier. Other calibration runs were occasionally recorded in the following months, either as a warm-up of the pilot or to update the decoder if required.

After the initial BCI calibration, closed-loop *online evaluation* sessions immediately followed. As before, the user was engaged in a cue-guided BCI control with a continuous visual feedback based on the output of the BCI decoder (Fig. 1a). During this phase, the classifier was re-calibrated four times (see Table 1) to better follow the evolution of the pilot’s brain patterns in the first two months of training, in which mutual learning is most likely to occur [34].

The third stage involved the pilot’s training in the actual *race control*. Initially, the pilot was asked to control the avatar only in the “left” and “right” turning sections, while relaxing in both the “noinput” and “headlight” sections. Afterwards, we included the possibility to deliver the “headlight” command as the close sequence of the two MI tasks. At every race control runs, we fully randomized the track sections.

The decision on when and whether to re-calibrate the decoder was taken by the operators evaluating the satisfaction of at least two of the following criteria: (i) if a change of the features location and/or band is identified from the user’s feature maps; (ii) if the classification accuracy drops below 75% during online evaluation sessions; (iii) if the pilot self-reports a difficulty in controlling the BCI output during either an online evaluation session or a race control session. The EEG data for the decoder re-calibration were taken through additional calibration runs. The features that were used in the previous decoder were selected also in the updated decoder, when possible. This approach was adopted to avoid abrupt changes of the classification model and to reinforce the stability of discriminant features in the long-term (e.g., C4 in the 20-22 Hz frequency bands, see Table 1).

After the Cybathlon BCI Series in September 2019, our pilot stopped the BCI training. In September 2020, after a year break, the training was resumed to prepare for the Cybathlon Global Edition. During this second training period, the pilot was almost fully engaged in the racing game, with no re-calibration of the BCI decoder with respect to the previous year. The pilot performed 2 calibration and 4 online runs in October 2020 for the final tuning of the BCI.

We approximately trained our pilot twice per week (three times in the weeks before the two Cybathlon events). Overall, our pilot performed 16 calibration, 30 online and 45 race runs before the 2019 Cybathlon BCI Series, and 3 calibration, 20 online and 68 race runs before the 2020 Cybathlon Global Edition. A schematic representation of the timeline of our pilot’s training protocol before the two competitions is reported in Fig. 1b.

### Data analysis and learning metrics

**Application performance.** Given the characteristics of the BCI race application, to measure the pilot’s performance we considered, as primary outcome, the time in seconds to complete a race track. In addition, we reported the time spent in each type of section (i.e., section crossing time). The average performance of the training sessions have been finally compared to the results obtained during the two competitions, to highlight the stability of our BCI system.

**BCI performance.** For these and the following analyses, we considered only EEG data in which no artifacts were detected by our EOG detector to ensure the results were not affected. The performance of the 2-class BCI system was measured by analysing the output of the Gaussian classifier, before the probability integration. In particular, we considered both the sample-by-sample accuracy (i.e., the percentage of correctly classified samples) and rejection (i.e., the percentage of samples whose classifier output is below the rejection threshold.) This second metric supplements the accuracy by providing an estimation of the system confidence in recognizing the correct class. The evolution of the user’s features was considered by analysing the discriminancy between the two MI tasks for each EEG channel-frequency pair. The class discriminancy was computed using the Fisher’s score as $FS = \frac{|\mu _1 - \mu _2|}{\sqrt{s_1^2 + s_2^2}}$, where $\mu _1$, $\mu _2$ are the means and $s_1$, $s_2$ the standard deviations of the PSD samples for class 1 (both hands) and class 2 (both feet), respectively. For the calibration and online runs, we exploited the cue associated with each trial for data labeling, while for the racing runs we considered and labeled the EEG data according to the logs provided by the BrainDriver application. Topographic discriminancy distributions were obtained by averaging the Fisher score of the PSD features in the $\beta$ frequency bands (16–26 Hz) corresponding to the brain rhythms modulated by our pilot (see Table 1).

**User learning.** To investigate the user learning, we decided to characterize the changes of his brain activity over the training periods. In particular, we analysed the evolution of the neural patterns associated with each mental task. Since a direct visualization of neural patterns’ trajectory in a high-dimensional neural space would be difficult to interpret, we identified two geometrical assessment metrics: (i) the between-class distance describes the discriminability between the brain activity of the two MI tasks (i.e., both hands - *bh*, both feet - *bf*) as

2 $$\begin{aligned} bcDist = \frac{\delta (\mu _{bh}, \mu _{bf})}{(\sigma _{bh}+\sigma _{bf})} \end{aligned}$$

It represents how well the different mental tasks can be discriminated in the neural space; (ii) the within-class distance measures the variation of the brain activity associated with each MI task *l* in the $r-th$ training run with respect to the first day of training, and is denoted by

3 $$\begin{aligned} wcDist = \frac{\delta (\mu _1^l, \mu _r^l)}{(\sigma _1^l+\sigma _r^l)} \end{aligned}$$

Despite not being directly related to a functional improvement of class discriminancy, the *wcDist* may still provide interesting information on the reorganization of brain network to optimize the execution of a specific mental task. In the above definitions of *bcDist* and *wcDist*, the numerator represents an opportune metric of distance between the mean neural patterns of the two classes (i.e., *bcDist*) or between two runs for a given class (i.e., *wcDist*), while the denominator computes the sum of the average distance around the means. For distance computation, two neural domains were considered, which are described below.

#### Channels’ domain

**Fig. 2:**
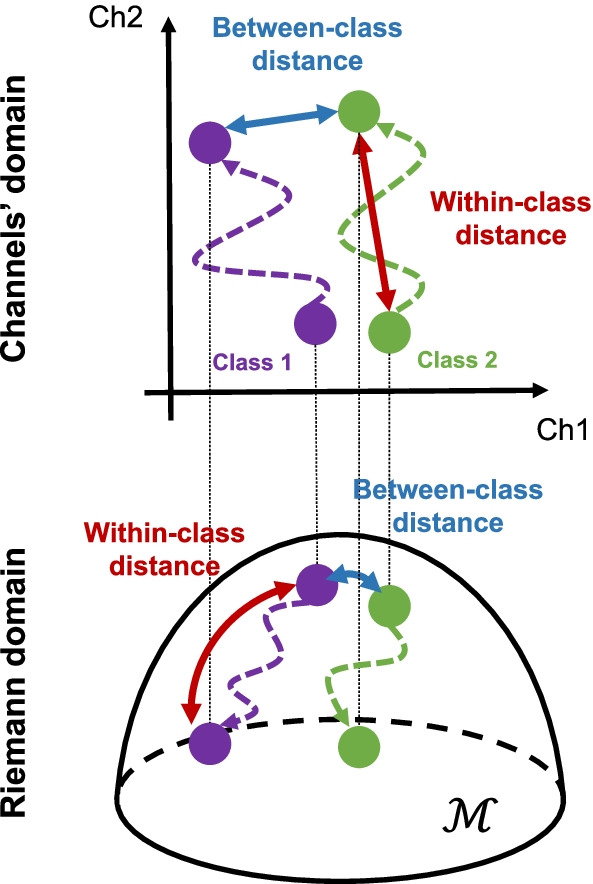
Schematic illustration of the metrics proposed to track user learning. The between-class distance represents the distance between the means of the EEG features distribution of the two motor imagery classes (i.e., both hands, both feet). The within-class distance is computed separately for the two classes as the distance of the means of the EEG features distribution with respect to the first day of training. In the channels’ domain the two metrics were calculated using the Euclidean distance, while in the Riemann domain we considered the geodesic distance (i.e., the shortest path between feature distributions following the Riemannian manifold ${\mathcal {M}}$)

The first is the domain of EEG channels. In particular, each axis corresponds to the PSD values of each of the 14 EEG electrodes over the sensorimotor cortex, averaged in the $\mu$ or $\beta$ bands. In this domain, the Euclidean distance was considered as distance metric between the arithmetic mean of two neural data distributions, as schematically illustrated in Fig. 2 (top). Thus, the computation of the *bcDist* corresponds to the Fisher’s score *FS*. For the *wcDist*, the Fisher’s score of each run was computed with respect to the first recorded run.

#### Riemann domain

In the second approach, we projected the EEG data of each channel in a different neural manifold based on Riemannian geometry. Let ${\mathbf {X}} \in {\mathbb {R}}^{C \times T}$, with *C* being the 14 zero-mean band-pass filtered EEG channels in the $\mu$ or $\beta$ bands, and *T* the number of samples in the 1 s-long window sliding every 62.5 ms. For the $i_{th}$ window, we computed the sample covariance matrix (SCM) as ${\mathbf {C}}_i = \frac{1}{T-1}{\mathbf {X}}_i{\mathbf {X}}_i^T$. These matrices are symmetric positive definite and lie in a $C \times C$ space. For each run, the geometric mean of the class-specific SCMs can be derived in the Riemann manifold using the algorithm proposed by [50]. According to Riemannian geometry, we can also obtain the distance between two covariance matrices as the geodesic distance of the two matrices in the Riemann manifold [51], as schematically illustrated in Fig. 2 (bottom). Given these definitions, the *bcDist* measures the distance between the SCM distributions associated with the two classes, while the *wcDist* between SCMs of each run with respect to the first run. More details on mean covariance matrix and geodesic distance computation in Riemannian geometry can be found in the Appendix.

### Statistical analysis

The effect of training on user’s performance has been evaluated by reporting Pearson’s correlation coefficients and their significance at the 95% confidence interval through Student’s t-test distribution between the performance metrics and the (chronological) race/run index. This analysis was carried out on race times, BCI accuracy, between-class and within-class distances. The same statistical test was used also to find significant correlations at the 95% confidence interval between the performance metrics. Additionally, inter-sessions improvements of these metrics were studied at the 95% confidence interval through nonparametric Kruskal-Wallis tests between the first and last 15 races/runs of both the 2019 and 2020 training periods. If the effect is deemed significant, Tukey-Kramer post-hoc tests were computed for multiple comparison analysis at the 95% confidence interval. Finally, the section crossing time improvements between the first and last 15 races are compared and tested for significant differences at the 95% confidence interval using unpaired, two-sided Wilcoxon nonparametric rank-sum tests.

## Results

### Application performance

**Fig. 3:**
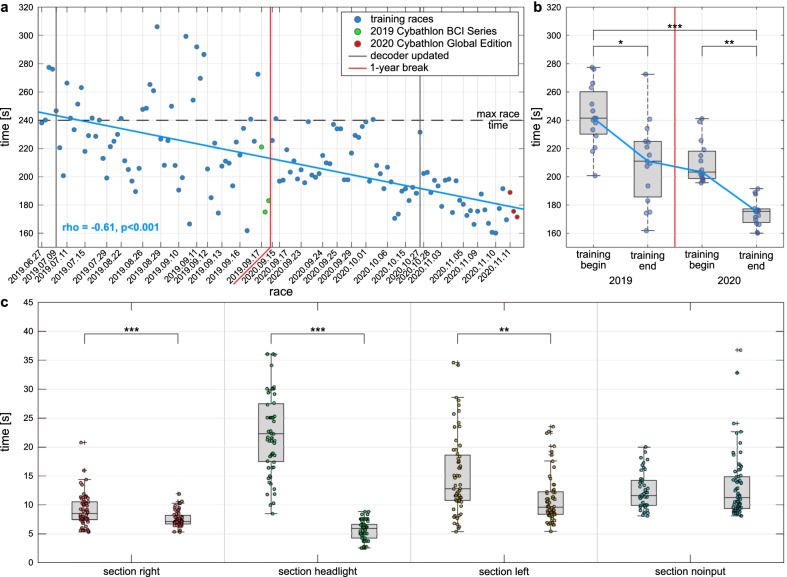
Cybathlon BCI race completion time. **a** Race completion times (s) achieved by our pilot throughout training. Training effect is shown by the linear fit and the Pearson correlation coefficient (significance tested with Student *t* test distribution). Dashed horizontal line illustrate the maximum race completion time allowed during the competition. Vertical thin lines indicate the date of each racing session, while vertical thick black lines represent the dates of decoder update. The break of 1 year is marked by a vertical red line. Markers colored in green and red show the race completion times obtained during the two competitions, in the 2019 BCI Series and the 2020 Global Edition respectively. **b** Boxplot of race completion times (s) in the first and last 15 races of 2019 and 2020 training periods. The box edges signify the 75th (top) and 25th (bottom) percentiles and the horizontal line the median of the corresponding distribution. The whiskers extend to the largest and smallest nonoutlier values. Single-race values are marked with filled circles. Statistically significant differences are shown with Tukey-Kramer post-hoc test. **c** Boxplot of section crossing time (s, time spent on each section) in the first 15 races of 2019 and the last 15 races of 2020 training periods. The box edges signify the 75th (top) and 25th (bottom) percentiles and the horizontal line the median of the corresponding distribution. The whiskers extend to the largest and smallest nonoutlier values. Outliers are marked with black crosses, while single-section values with filled circles. Statistically significant differences are shown with two-sided Wilcoxon ranksum tests. * $p<.05$, ** $p<.01$, *** $p<.001$

The time performance of our pilot in the final application are reported in Fig. 3. The negative Pearson’s correlation between race time and race index highlights a statistically significant effect of training on race performance (Fig. 3a, $r=-0.61$, $p<.001$, $N=133$). Indeed, the pilot was able to reduce the race completion time from $242.89 \pm 21.64$ s in the first 15 runs to $209.93 \pm 29.11$ s in the last 15 runs in 2019 (mean ± standard deviation) (Fig. 3b, $p<.05$, Tukey-Kramer post-hoc test), well far away from the maximum allowed race time of 240 s. Interestingly, no significant difference has been found between the last 15 runs in 2019 and the first 15 runs in 2020 (Fig. 3b, $p=.99$), showing stable performance even after a year. An additional reduction of race completion time to $174.41 \pm 9.44$ s is then visible in the last 15 runs in 2020, including the day of the final competition. This improvement is also found to be statistically significant (Fig. 3b, $p<.001$). To better understand the pilot’s performance, Fig. 3c reports the comparison of the section crossing time for each type of sections between the first and last 15 runs. Overall, our pilot was able to reduce the time spent in each type of active sections. Statistically significant improvements have been found for the right sections ($p<.001$, two-sided Wilcoxon ranksum test), headlight sections ($p<.001$) and left sections ($p<.01$), spending on average less than 10 s in all of them after training. No significant improvement has been found for the “noinput” sections ($p=.93$).

### Competition outcomes

Even if the improvements of our pilot previously reported are an important sign of the efficacy of the human-machine interaction that was established during the training periods, an unequivocal proof of the BCI performance is shown by the results obtained during the two Cybathlon events. In fact, these events represent a unique opportunity to measure the translational efficacy of the BCI system in non-ideal conditions (e.g., out-of-lab, in front of audience), similar to the ones that would be faced in a daily usage. The first competition was held in a conference hall during the “8th Graz Brain-Computer Interface Conference”. Two races were performed during a qualification phase, and the four teams with the lowest race time participated in the one-hot final race. The official results of the 2019 Cybathlon BCI Series are reported in Table 2. Our pilot qualified with a time of 175 s, setting the competition record, and won the final with a time of 183 s. The 2020 Cybathlon Global Edition was instead performed remotely, due to the COVID-19 health emergency. The competition consisted of three consecutive races, and the three pilots who obtained the lowest race completion time won the gold, silver and bronze medal, respectively. The official results of the competition for the BCI discipline are reported in Table 2, in which our pilot achieved, for the second time, the best race performance of 172 s.

**Table 2:** Cybathlon BCI race results

2019 Cybathlon BCI Series							
Rank	Team	Final		Q2		Q1	
		Distance [m]	Time [s]	Distance [m]	Time [s]	Distance [m]	Time [s]
1	WHI Team	500.0	183	500.0	175	500.0	221
2	MIRAGE 91	500.0	229	500.0	215	492.7	240
3	NeuroCONCISE	386.6	240	455.5	240	296.4	240
4	Mahidol BCI	99.9	57	500.0	233	462.0	240
5	NITRO 1	399.8	240	422.8	240	421.0	240
6	NITRO 2	390.5	240	435.8	240	418.4	240

2020 Cybathlon Global Edition							
Rank	Team	Race 1		Race 2		Race 3	
		Distance [m]	Time [s]	Distance [m]	Time [s]	Distance [m]	Time [s]
1	WHI Team	500.0	189	500.0	176	**500.0**	**172**
2	Mahidol BCI	500.0	208	500.0	186	**500.0**	**176**
3	Neurorobotics	500.0	224	**500.0**	**213**	500.0	237
4	SEC FHT	354	240	500.0	235	**500.0**	**221**
5	MIRAGE 91	440	240	452	240	**500.0**	**223**
6	NeuroCONCISE	439	240	**452**	**240**	350	206
7	Phoenix	**439**	**240**	437	240	496	240

The table presents the race completion times, distance, and ranking of all competing teams during the two Cybathlon competitions. The 2019 Cybathlon BCI Series was organized in two qualification races (Q1, Q2) and a Final race with the best four teams. The 2020 Cybathlon Global Edition consisted in three independent races and the result of the best race of each team (in bold) was considered for the ranking

### BCI performance

**Fig. 4:**
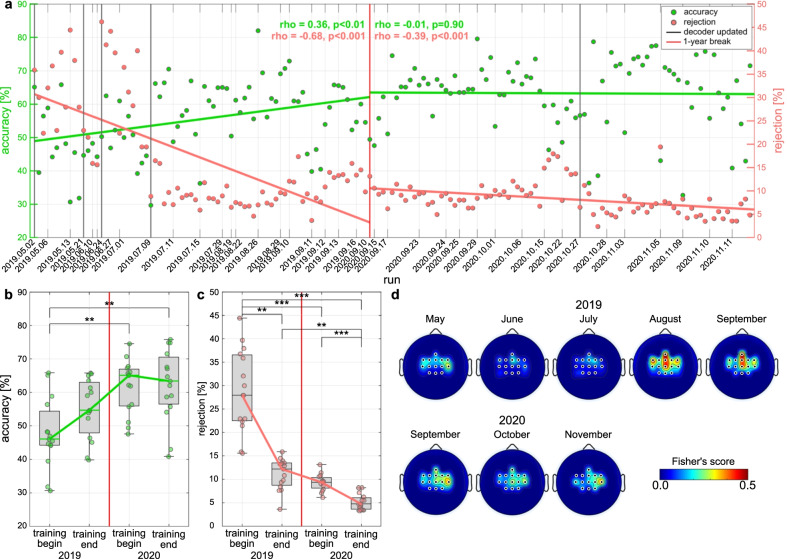
BCI performance and topographic maps. **a** Evolution over training runs of the decoder accuracy (green, % of correctly classified samples) and rejection (red, % of samples whose prediction was discarded due to low confidence). Their corresponding linear fits and Pearson correlation coefficients (significance tested with Student *t* test distribution) were evaluated for the two years (2019, 2020) separately. Vertical thin lines indicate the date of each training session, while vertical thick black lines represent the dates of decoder update. The break of 1 year is marked by a vertical red line. (b-c) Boxplots of decoder accuracy **b** and rejection **c** in the first and last 15 runs of 2019 and 2020 training periods. The box edges signify the 75th (top) and 25th (bottom) percentiles and the horizontal line the median of the corresponding distribution. The whiskers extend to the largest and smallest nonoutlier values. Single-run values are marked with filled circles. Statistically significant differences are shown with Tukey-Kramer post-hoc tests. **d** Topographic maps of discriminancy per training month on the 14 EEG channel locations over the sensorimotor cortex. Bright color indicates high discriminancy between Both Hands and Both Feet MI tasks. The discriminancy of each channel is quantified as the Fisher score of the EEG signal’s power spectral density distributions for the two mental classes in the $\beta$-band (16–26 Hz) within each run. Each map illustrates local Fisher scores (with interchannel interpolation) averaged over all runs within the month

The performance of the BCI system has been analysed as the capability of discriminating the two mental tasks (i.e., hands vs. feet MI) from the user’s EEG signals. Fig. 4a shows the evolution of the BCI performance in terms of percentage of correctly classified samples and percentage of rejected samples. For this analysis we considered also the online runs together with the races. Significant positive/negative Pearson’s correlations between accuracy/rejection and run index in 2019 ($r=0.36/-0.68$, $p<.01/.001$, $N=75/75$) revealed the success of our mutual learning training strategy in increasing the overall classification performance of the BCI system. In particular, a significant reduction of $17.8\%$ in rejection (Fig. 4b, $p<.01$, Tukey-Kramer post-hoc test) have been obtained from the first to the last runs of the 2019 training period. The pilot was capable of reducing the percentage of rejected samples also in 2020 (Fig. 4a, $p<.001$), with a significant linear correlation to run index ($r=-0.39$, $p<.001$, $N=88$). No statistically significant difference in rejection have been found between the end of the 2019 and the beginning of 2020 (Fig. 4c, $p=.78$). Remarkably, the accuracy increased by $7.4\%$ after the year off, even if not statistically significant (Fig. 4b, $p=.24$). Fig. 4d portraits the arising of discriminant SMRs patterns ($\beta$-band, 16–26 Hz) which are coherent with the selected BCI paradigm. The pilot started in May with a higher activation of EEG features associated with the both hands MI (lateral, electrodes FC3, C3, FC4, C4 of the 10–20 EEG system). Likely related to the longitudinal training, he also acquired emerging features associated with both feet MI (medial, electrodes Fz, FCz, Cz), which led to the significant increase of accuracy in September 2019. Similar topographies between the end of 2019 and the beginning of 2020 highlight the robustness of the BCI skills acquired by the pilot and the effectiveness of our training strategy in the long-term.

### User learning

**Fig. 5:**
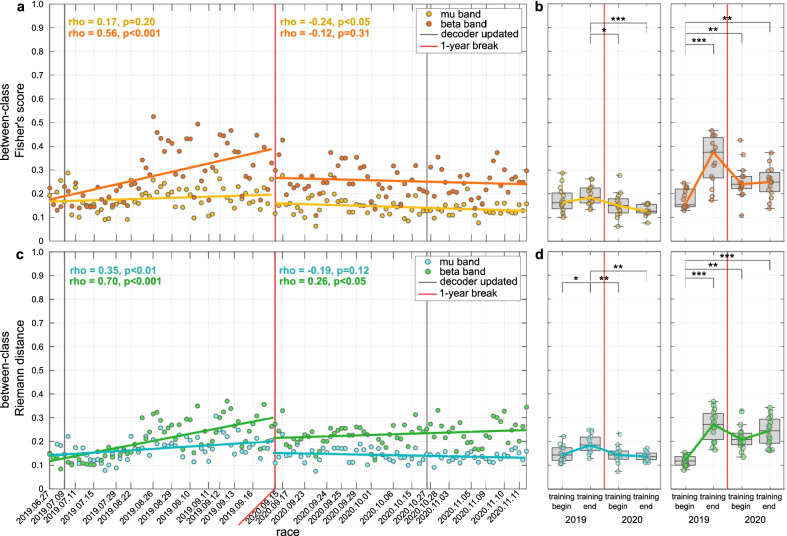
Between-class distance in channels’ and Riemann domains. **a**–**c** Evolution over races of the between-class distance in channels’ domain **a** and Riemann domain **c** computed in the $\mu$-band (8–12 Hz) and $\beta$-band (16–26 Hz). Their corresponding linear fits and Pearson correlation coefficients (significance tested with Student *t* test distribution) were evaluated for the two years (2019, 2020) separately. Vertical thin lines indicate the date of each racing session, while vertical thick black lines represent the dates of decoder update. The break of 1 year is marked by a vertical red line. **b**–**d** Boxplots of between-class distance in channels’ domain **b** and Riemann domain **d** for $\mu$-band (left) and $\beta$-band (right) in the first and last 15 runs of 2019 and 2020 training periods. The box edges signify the 75th (top) and 25th (bottom) percentiles and the horizontal line the median of the corresponding distribution. The whiskers extend to the largest and smallest nonoutlier values. Single-run values are marked with filled circles. Statistically significant differences are shown with Tukey-Kramer post-hoc tests

The evolution of user learning has been evaluated by analysing the modification of the user’s neural patterns in two multi-dimensional domains, corresponding to the channels’ domain and the Riemann manifold. Fig. 5 shows the evolution of the between-class distance for the $\mu$ and $\beta$ bands in the channels’ domain and in the Riemann domain. A significant positive increase of discriminancy in $\beta$-band is visible in both domains (channels/Riemann) at the end of 2019 ($p<.001/.001$, Tukey-Kramer post-hoc tests), showing a significant linear correlation with training ($r=0.56/0.69$, $p<.001/.001$, $N=61/61$) which is then kept stable in the long-term. The Riemann domain displays also a positive increase of *bcDist* for the $\mu$-band in 2019 (Fig. 5c, $r=0.38$, $p<.01$, $N=61$), even if lower than for the $\beta$-band. On the other hand, no significant evolution of the *bcDist* happened for the $\mu$-band in the channels’ domain, which is consistent with our selection of $\beta$ features to train the classifier (see Table 1). Again, no statistically relevant variation can be appreciated in $\beta$ band between the training end in 2019 and the training begin in 2020, which is consistent with the BCI and application performance.

**Fig. 6:**
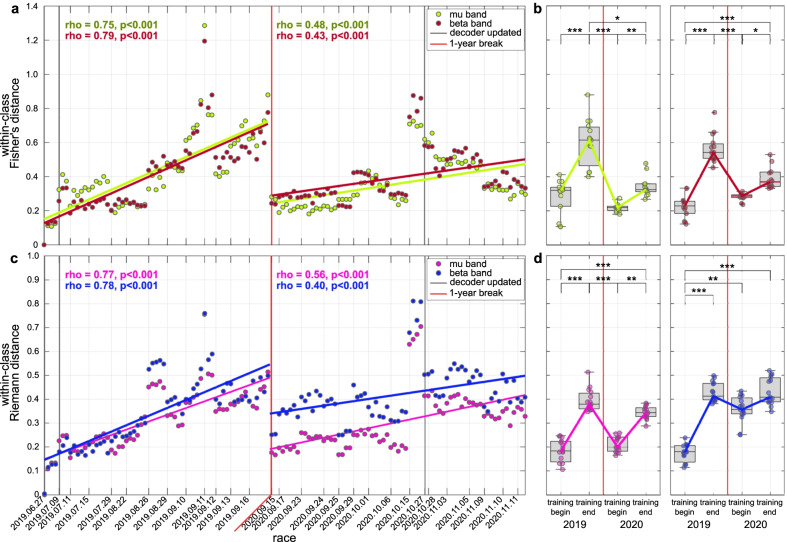
Within-class distance in channels’ and Riemann domains. **a**–**c** Evolution over races of the within-class distance in channels’ domain **a** and Riemann domain **c** computed in the $\mu$-band (8–12 Hz) and $\beta$-band (16–26 Hz). Their corresponding linear fits and Pearson correlation coefficients (significance tested with Student *t* test distribution) were evaluated for the two years (2019, 2020) separately. Vertical thin lines indicate the date of each racing session, while vertical thick black lines represent the dates of decoder update. The break of 1 year is marked by a vertical red line. **b**–**d** Boxplots of within-class distance in channels’ domain **b** and Riemann domain **d** for $\mu$-band (left) and $\beta$-band (right) in the first and last 15 runs of 2019 and 2020 training periods. The box edges signify the 75th (top) and 25th (bottom) percentiles and the horizontal line the median of the corresponding distribution. The whiskers extend to the largest and smallest nonoutlier values. Single-run values are marked with filled circles. Statistically significant differences are shown with Tukey-Kramer post-hoc tests

The evolution of the second user learning metric, the within-class distance, is depicted in Fig. 6 for the channels’ domain and the Riemann domain. This metric aims at highlighting changes of the user’s brain activity as a consequence of longitudinal training with our BCI. For the *wcDist*, we reported the distance averaged across the two classes. For completeness, the *wcDist* of the two classes separately is provided in the Additional files (Additional file 4: Fig. S4 and Additional file 5: Fig. S5). The effect of the initial training (in 2019) on the user’s neural patterns is demonstrated in both domains (channels/Riemann) by a significant positive Pearson’s correlation of the *wcDist* in the $\beta$-band with race index ($r=0.77/0.78$, $p<.001/.001$, $N=61/61$). The user shows a significant increase of the *wcDist* in the $\mu$-band ($r=0.75/0.75$, $p<.001/.001$, $N=61/61$), even if it was not considered in the feature selection for our pilot’s decoder. A significant distance of his neural patterns has been found close to the 2019 BCI Series competition with respect to the first training runs in both frequency bands ($\mu$: $p<.001/.001$, $\beta$: $p<.001/.001$, Tukey-Kramer post-hoc tests). Interestingly, the representation in the channels’ domain shows a regression of the pilot’s brain activity to its original state after the 1-year break. Indeed, the *wcDist* of the user at the beginning of 2020 results to be significantly decreased compared to the end of 2019 (Fig. 6b, $\mu$: $p<.001$, $\beta$: $p<.001$), with neural patterns similar to the ones at the first days of training ($\mu$: $p=0.27$, $\beta$: $p=0.47$). By training again, the *wcDist* increases in the 2020 as well (Fig. 6a, $r=0.48/0.43$, $p<.001/.001$, $N=72/72$), with comparable values between the days close to the two competitions in the $\beta$ band ($p=0.15$).

Conversely, it is worth to highlight the behavior of the within-class distance in the Riemann space. Our analysis revealed two different behaviors for the trained $\beta$-band and the untrained $\mu$-band (Fig. 6c–d). In particular, the latter is characterized by the same discontinuity after the 1-year break as in the channels’ domain, with a significant reduction of the distance at the beginning of 2020 ($p<.001$) towards the values of the training begin in 2019 ($p=0.90$). On the other hand, the *wcDist* in the Riemann domain presents no differences in $\beta$-band between the training end in 2019 and the training begin in 2020 ($p=0.12$), and remained stable until the day of the main competition ($p=0.98$).

## Discussion

In the present study, we explored a unique dataset registered during the training for the participation to the Cybathlon BCI race competitions in 2019 and 2020. This dataset contains EEG data from more than 40 training sessions with an end-user engaged with our MI BCI system. Moreover, it gave us the opportunity to test the robustness of the user-decoder interaction reinforced by the training in the long term (1 year) without using a BCI, which represents an unprecedented opportunity in the field.

### Mutual learning in BCI

As shown in Fig. 4a, the first months of training were characterized by a rapid increase of classification accuracy and confidence in discriminating between the two mental tasks, particularly in the first three months of training (May-July 2019). These results are in line with well-established evidences that the concomitant training of the two main actors (i.e., user and decoder) improves BCI performances already after a few sessions of practice, and that they positively correlate with improved application performances (Additional file 2: Fig. S2) [34]. Even if the concept of mutual learning is becoming more and more popular in the BCI community, also thanks to events like the Cybathlon competitions, the role of user learning in this process is often overlooked. Previous studies focused mostly on the machine learning aspects, with the most common approach to the mutual learning consisting in regularly adapting the decoder parameters to deal with the intra- and inter-session variability of the user’s brain patterns [35–38]. However, a more or less frequent re-calibration of the BCI system could potentially mask the effect of pure user learning on the performance [20].

In light of these considerations, we adopted a different strategy. On the one hand, we opted for a game control paradigm which allowed the use of a simpler 2-class BCI decoder to implement the four game control commands. Indeed, it is well known that the BCI classification performance drops sharply as the number of classes to be decoded increases. Since the BCI represents a difficult task in itself, a multi-class classification approach could have been a task too challenging for the pilot, with the risk of hampering the user learning [52]. On the other hand, we decided to update the BCI system only a few times to leave more degrees of freedom to the user. This strategy was corroborated by previous experience and by the high accuracy of our BCI system [34], entailing several advantages. Firstly, it allowed to significantly reduce the time spent in tedious calibration runs and to train our pilot in self-modulating his brain rhythms as soon as possible in the BrainDriver game. Indeed, a closed-loop BCI usage in the final application have shown to significantly boost the training effect [34, 38]. Secondly, a BCI system that is too adaptive to the user could have compromised the learning process of our pilot by overly relying on the system capabilities rather than on his progress [20]. Conversely, it is worth highlighting that we last updated the decoder at the beginning of July 2019 and we made no re-calibration until October 2020. On the first day of the 2020 training period (i.e., 2020/09/15), the pilot directly used the decoder that was previously calibrated on 2019/07/09, more than 1 year earlier. This fact ensures that the visible and significant improvements of both BCI and race performances in between this period can only be traced back to modifications of user’s ability in correctly modulating his SMRs while receiving training.

The stability of BCI and application performances of our pilot (Fig. 3, 4) demonstrates that, once learned, he was capable of recalling the BCI skills accurately and consistently even after a 1-year break. However, how the brain achieves this behavioral stability is an open question. Improvements in online classification accuracy and/or application performance do not necessarily imply changes at the brain level underpinning BCI learnability [53]. Hence, the main contribution of this work is the provision of neural correlates associated with user learning of BCI skills and their evolution over time.

### User learning in channels’ domain

The first attempt to investigate the evolution of the user’s brain activity concerned the analysis of the EEG features distribution used to create the decoder (i.e., the channels’ domain) since it directly reflects the ability of the user to control the BCI. In particular, the emergence of SMRs modulations associated with the mental tasks is the most commonly used index in the BCI literature to monitor longitudinal improvements [14, 15, 34–36, 54]. Except for [34], none of these studies explicitly related the evolution of SMRs modulations to consistent and continuous changes of BCI performance. Herein, we reported a strong correlation between classification accuracy and *bcDist* in $\beta$ band, but not in $\mu$ band (Additional file 3: Fig. S3a), establishing the impact of operant conditioning training in user learning. Indeed, since the system output—thus the feedback to the user—depends solely on the features selected during decoder calibration, we expected our pilot to improve the between-class distance only in the frequency band for which he received a coherent feedback during training (Fig. 4d) [55].

Despite its straightforwardness, the sole neuroimaging-based difference of class prototypes is likely to provide only a partial picture of the neural processes occurring in the user’s brain as a consequence of training. The time-dependent variability of the brain signals may lead to within-class modifications of neural patterns, which are not directly related to maximizing class separability. We found that the brain activity of our pilot during the execution of the mental tasks significantly shifted with regular training (Fig. 6a). Remarkably, this shift interested the two classes symmetrically (Additional file 4: Fig. S4), and both the $\mu$ and $\beta$ bands (Fig. 6b), indicating a more holistic phenomenon which is not limited to class-specific discriminant features. While an increase of the within-class distance is usually seen negatively in the literature—and a lot of effort is spent in the development of machine learning algorithms to minimize it [14, 36]—recent literature promotes the idea that it could be linked with a positive user adaptation to the BCI system [20, 35]. The user modifies his brain activity to produce brain signal modulations that match more closely those expected by the decoder [35]. However, the analysis of the *wcDist* in the channels’ domain reported in Fig. 6a, b shows that these modifications are limited to transitory effects which occur only when the user is regularly receiving training. These results are not consistent with the continuous improvements shown by our pilot, substantiated by the lack of correlation between the *wcDist* in the channels’ domain and the classification accuracy (Additional file 3: Fig. S3c). This could mean that this representation shows only short-term adjustments of the user’s BCI aptitude, rather than a stable learning effect in the long term.

### User learning in neural manifold

As previously discussed and widely established in the literature, the analysis of the EEG signals in the channels’ domain is effective and convenient for the real-time classification of mental tasks, but it fails in capturing the long-term stability of the BCI skills demonstrated by our pilot. In this paper, we overcome this limitation by proposing to investigate user learning in an hyperspace—the Riemann manifold—different with respect to the input space generated by the features that are exploited in the BCI system.

From the perspective of class discrimination (Fig. 5), our results show that the between-class distance in the Riemannian geometry matched the performance obtained in the channels’ input space. Like before, a strong correlation between classification accuracy and the Riemann *bcDist* in $\beta$-band, but not in $\mu$-band, was found (Additional file 3: Fig. S3b). This finding was not completely unexpected since the use of the signal covariance matrices as features of interest for classification of mental tasks is becoming increasingly popular in BCI applications [56–59] and they have been also exploited by a team during the Cybathlon competitions [35]. Comparably to the channels’ domain, regular training induced a sharp shift of the within-class EEG covariance matrices as our pilot was adapting to the use of the BCI system in the 2019 (Fig. 6c). Nevertheless, of greater interest is the different evolution in the Riemann domain of the *wcDist* between the two frequency bands after the one year break. While the *wcDist* in the $\mu$ band significantly decreased from 2019 to 2020, the $\beta$ band did not show this regression and remained stable between the two years (Fig. 6d and Additional file 5: Fig. S5). Thanks to these findings, we pinpoint the existence and definition of two type of neural modifications as a consequence of user learning: the first are short-term modifications, that spontaneously arise during training since they involve also features which are not directly targeted by the training (i.e., $\mu$ band). Given these characteristics, these modifications are likely to be not robust enough to endure for a long between-session period; but they may be at the basis of the mutual-learning process and of the emergence of new SMRs features which were not considered in the previous decoders [20, 34]. On the other hand, the unique stability of the *wcDist* in the Riemann domain of the $\beta$ band—whose features were consistently selected and reinforced over the all training period (Table 1)—may underpin stable modifications of our pilot’s brain activity related to a long-term learning of the BCI skills which allowed him to maintain his excellent performance even without a continuous training and/or re-calibration of the decoder. In support to this statement, a strong positive correlation between classification accuracy and $\beta$-band *wcDist* in the Riemann domain is reported (Additional file 3: Fig. S3d), which would not have been found by limiting the analyses to the channels’ domain only.

We believe that the results of this paper open a new way of studying and analysing user learning in BCI, breaking the typical approach of searching learning correlates only in the space spanned by the neural features used as input to the decoder. A similar approach is actually commonly considered to enhance the understanding of BCI training in stroke rehabilitation: neural mechanisms underlying the clinical effects of BCI therapy are often evaluated through various markers which are not limited to a stronger desynchronized activity during MI tasks; they include also interhemispheric imbalance, functional connectivity changes [60–62], and even functional and structural assessments through different neuroimaging techniques (e.g., functional near-infrared spectroscopy (fNIRS), functional magnetic resonance imaging (fMRI)) [63, 64]. In this line, the evolution of the *wcDist* in the Riemann domain may be helpful to better follow the progress of user learning of BCI skills together with conventional metrics (e.g., classification accuracy). It is interesting to note an evident discontinuity of the Riemann’s *wcDist* in the runs right before our decision of re-calibrating the decoder in October 2020 (Fig. 6c). Even if further analysis would be required to support this hypothesis, an abrupt changeover of the user’s neural state in performing the mental tasks could hide a break of the user-decoder interaction, wandering in an area of the neural manifold that prevented our pilot from efficiently controlling the BCI. We thus suggest, and better investigate in future studies, that a monitoring of the proposed Riemann’s within-class distance may hint when updates of the decoder parameters are required.

Overall this study confirms the effective learnability of BCI thanks to longitudinal usage, i.e. increase of classification accuracy across the training sessions [34–38]. In addition, this study shows that by focusing the training strategy on improving user learning, it is feasible to achieve a robust stabilization of BCI skills and features over a long period of time. An hypothesis that might explain these results is that the regular user-centered BCI training applied by us induced a functional reorganization of our pilot’s neural networks that are responsible for the imagination of the two motor tasks. This hypothesis is supported by previous studies [62, 63, 65] revealing that BCI-guided rehabilitation training induces long-term neuroplasticity modulations which are kept up to 6 months after the intervention [63]. A similar phenomenon was previously seen in invasive BCI experiments, which showed that the brain is capable of encoding a stable representation of motor-related tasks for very long periods of time (i.e., from months up to years) [66–68]. However, in this work we show for the first time in a non-invasive BCI application the possibility to achieve a stability of BCI skills through learning for a time period longer than 1 year of non-use of the BCI. This feature allowed our pilot to retain his performance at the beginning of the 2020 training period without the need of any decoder re-calibration. In future work, we will perform a more in depth analysis in relation to connectivity to identify if the seen stable performance of our pilot were due to the effective existence of a functional reorganization or to the adoption of a more efficient motor imagery strategy activating existing neural networks. Nevertheless, we believe that our findings—combined with the successful results obtained in the challenging scenarios of the Cybathlon competitions—may provide a strong contribution in shifting the focus of the BCI community: not only to the machine learning of the decoder, but also in investigating novel training procedures to boost the user learning and the mutual adaptation of the user to the BCI system.

### Limitations

There are certain limitations of this study that need to be mentioned. The major limitation concerns the inclusion of only one subject in the dataset which holds us back from drawing definitive conclusions. Thus, in future work we aim at recruiting a larger cohort of users in a longitudinal BCI training in order to strengthen the preliminary results on user learning reported in this paper. Nevertheless, as previously mentioned, the type of training and the limited number of re-calibrations assure us that there has certainly been a learning process in our pilot, and that the significant improvements in performance over time are mainly due to this.

The second limitation to be mentioned is that the study was conceived to be observational and uncontrolled. Still, the results obtained by our competitors may be helpful as a fair control group to highlight the effective importance of user learning in BCI. Indeed, the other teams adopted a training strategy focused on frequent re-calibrations of their decoders [35–38]. Except for [36], all the other teams reported a significant worsening of their pilots’ performances during the competitions, explained by the presence of audience or, in general, conditions of stress. It is well-known that the psychophysiological state of the user has a negative effect on BCI operation [69–71]. Interestingly, our pilot was able to achieve race completion times during the competitions that were comparable or even better than his average results in the previous days. The fact that our pilot was already used to competitions surely represents an important factor accounting for these results. Nevertheless, we speculate that the stable user learning attained by our pilot, identifiable both at the behavioral and neural level, strongly contributed to the achievement of not only a high BCI accuracy, but more importantly a high reliability which represents a critical challenge in the field.

## Conclusions

This paper presents the analysis of a unique longitudinal study that allowed us to deepen our understanding on user learning during long-term BCI training in preparation to the Cybathlon competition, spanning more than 1 year. Our results undoubtedly demonstrated that our pilot effectively learnt how to control the BCI. As main contribution, herein we proposed a multifaceted perspective on the evolution of user learning, enriching the information gathered through conventional metrics (e.g., BCI accuracy, features’ topographic distribution) by investigating novel neural correlates of learning. We revealed that examining the neural patterns associated to the BCI tasks in the Riemann space provides valuable insights to explain the progressive improvements of our pilot’s performance in the final application, as well as the stability of his BCI skills, even if not using the system for a long period. In future work, such approach could be used to monitor the co-adaptation of the user-decoder dyad and potentially provide a marker predicting when a re-calibration of the decoder is required.

### Supplementary information

**Additional file 1.** Schematic representation depicting the design of the implemented game control paradigm. Both hands (BH) and both feet (BF) BCI commands were associated with the delivery of “left” (L) and “right” (R) game commands, respectively. The third active command, the “headlight” (H) was instead obtained through a sequential combination approach, by sending two different BCI commands within a configurable time window (e.g., 2 seconds). While no BCI commands are generated, the pilot is considered resting and the game receives the “noinput” (N) command.

**Additional file 2.** Relationship between our pilot’s race completion times and the classification accuracy of the decoder. Higher decoding performance generally corresponds to a shorter racing time, as shown by the linear fit and the Pearson correlation coefficient (significance tested with Student t test distribution).

**Additional file 3.** Relationship between the classification accuracy of the decoder and the between-class distance **a**, **b** and within-class distance **c**, **d** of our pilot’s EEG features. Linear fit and the Pearson correlation coefficients (significance tested with Student t test distribution) show a positive correlation between accuracy and band between-class distances in both the channels’ domain (**a**) and Riemann domain (**b**). A positive correlation between accuracy and the band within-class distance was found only in the Riemann domain (**d**), but not in the channels’ domain (**b**).

**Additional file 4.** Evolution over races of the within-class distance in channels’ domain for the both hands class (top) and for the both feet class (bottom) in the μ-band (8–12 Hz) and β-band (16–26 Hz). Their corresponding linear fits and Pearson correlation coefficients (significance tested with Student t test distribution) were evaluated for the two years (2019, 2020) separately. Vertical thin lines indicate the date of each racing session, while vertical thick black lines represent the dates of decoder update. The break of 1 year is marked by a vertical red line.

**Additional file 5.** Evolution over races of the within-class distance in Riemann domain for the both hands class (top) and for the both feet class (bottom) in the μ-band (8–12 Hz) and β-band (16–26 Hz). Their corresponding linear fits and Pearson correlation coefficients (significance tested with Student t test distribution) were evaluated for the two years (2019, 2020) separately. Vertical thin lines indicate the date of each racing session, while vertical thick black lines represent the dates of decoder update. The break of 1 year is marked by a vertical red line.

## Data Availability

The dataset analysed during the current study is available from the corresponding authors upon reasonable request.

## Appendix

### Riemann geodesic distance

The Riemannian space is a differentiable manifold in which the tangent space at each point is a finite dimensional Euclidean space. For each point ${\mathbf {C}}$ (i.e. symmetric positive-definite (SPD) matrix) in the manifold ${\mathcal {M}}$, a tangent Euclidean space ${\mathcal {T}}_{{\mathbf {C}}}{\mathcal {M}}$ can be defined which is locally homomorphic to the manifold. Given this definition and letting *C*(*n*) be the set of all possible $n \times n$ SPD matrices, the distance between two points ${\mathbf {C}}_1, {\mathbf {C}}_2 \in C(n)$ can be computed as the length of the shortest curve between these two points along the manifold, also called geodesic distance, which is denoted as

4 $$\begin{aligned} \delta _R({\mathbf {C}}_1, {\mathbf {C}}_2) = ||log({\mathbf {C}}_1^{-1}{\mathbf {C}}_2)||_F = [\sum _{i=1}^{n}{log^2 \lambda _i}]^{1/2} \end{aligned}$$

where $||\cdot ||_F$ is the Frobenius norm, and $\lambda _i, i=1,...,n$ the real eigenvalues of ${\mathbf {C}}_1^{-1}{\mathbf {C}}_2$. The Riemannian geodesic distance is characterized by the following main properties:

- $$\delta _R({\mathbf {C}}_1, {\mathbf {C}}_2)=\delta _R({\mathbf {C}}_2, {\mathbf {C}}_1)$$
- $$\delta _R({\mathbf {C}}_1^{-1}, {\mathbf {C}}_2^{-1})=\delta _R({\mathbf {C}}_1, {\mathbf {C}}_2)$$
- $$\delta _R({\mathbf {W}}^T{\mathbf {C}}_1{\mathbf {W}}, {\mathbf {W}}^T{\mathbf {C}}_2{\mathbf {W}})=\delta _R({\mathbf {C}}_1, {\mathbf {C}}_2) \forall \, {\mathbf {W}} \in G(n)$$

with *G*(*n*) the set of all possible $n \times n$ invertible matrices.

### SCM mean in Riemann geometry

The mean value represents the simplest statistical descriptor of a distribution of points in the space. Given a set of *K* SPD matrices ${\mathbf {C}}_1, ..., {\mathbf {C}}_K \in C(n)$, their arithmetic mean ${\mathcal {C}}_E$ could be computed in the Euclidean space as the SPD matrix ${\mathbf {C}}$ that minimizes the sum of squared Euclidean distances $d_E(\cdot ,\cdot )$

5 $$\begin{aligned} {\mathcal {C}}_E = arg \min _{{\mathbf {C}} \in C(n)} \sum _{k=1}^{K}{d_E^2({\mathbf {C}}_k,{\mathbf {C}})} = \frac{1}{K}\sum _{k=1}^{K}{{\mathbf {C}}_k} \end{aligned}$$

This arithmetic mean can be used as a reference point in the manifold where the tangent space is computed [72].

Similarly, we can compute the geometric mean of the SPD matrices in the Riemannian manifold using the definition of distance in (4) as

6 $$\begin{aligned} {\mathcal {C}}_R = arg \min _{{\mathbf {C}} \in C(n)} \sum _{k=1}^{K}{\delta _R^2({\mathbf {C}}_k,{\mathbf {C}})} \end{aligned}$$

This geometric mean is proven to always exist and to be unique for a finite set of points [50]. However, since for $K>2$ a closed-form expression is not possible, several methods have been proposed to solve this problem iteratively [73]. In this study, we computed the geometric mean with the following algorithm:

1. Initialize the tangent space in the reference point computed using (5);
2. Project the SPD matrices in the tangent space and compute the closed-form arithmetic mean in this Euclidean space;
3. Project the arithmetic mean back in the manifold (i.e., estimated geometric mean ${\mathcal {C}}_R$);
4. Compute the new tangent space using ${\mathcal {C}}_R$ as reference point;
5. Repeat steps 2–4 until convergence.

The full implementation of the above algorithm can be found in [50], while for a more detailed explanation of the manifold-to-tangent and tangent-to-manifold projection functions we refer the reader to [72].

## Abbreviations

BCI: Brain-computer interface
CVA: Canonical variate analysis
EEG: Electroencephalography
EMG: Electromyogram
EOG: Electrooculography
MI: Motor imagery
MRC: Medical Research Council
PSD: Power spectral density
ROS: Robot operating system
SCM: Sample covariance matrix
SMR: Sensorymotor rhythm
SOM: self-organizing maps
SPD: Symmetric positive-definite
SSVEP: Steady state visual evoked potential

## Footnotes

1 https://cybathlon.ethz.ch/en/event/disciplines/bci

2 https://github.com/rosneuro

3 https://github.com/rosneuro
