# Supplementary figures and images for: Neural correlates of user learning during long-term BCI training for the Cybathlon competition
Source: J Neuroeng Rehabil. 2022 Jul 5;19:69. doi: 10.1186/s12984-022-01047-x (PMC9254548; doi:10.1186/s12984-022-01047-x)

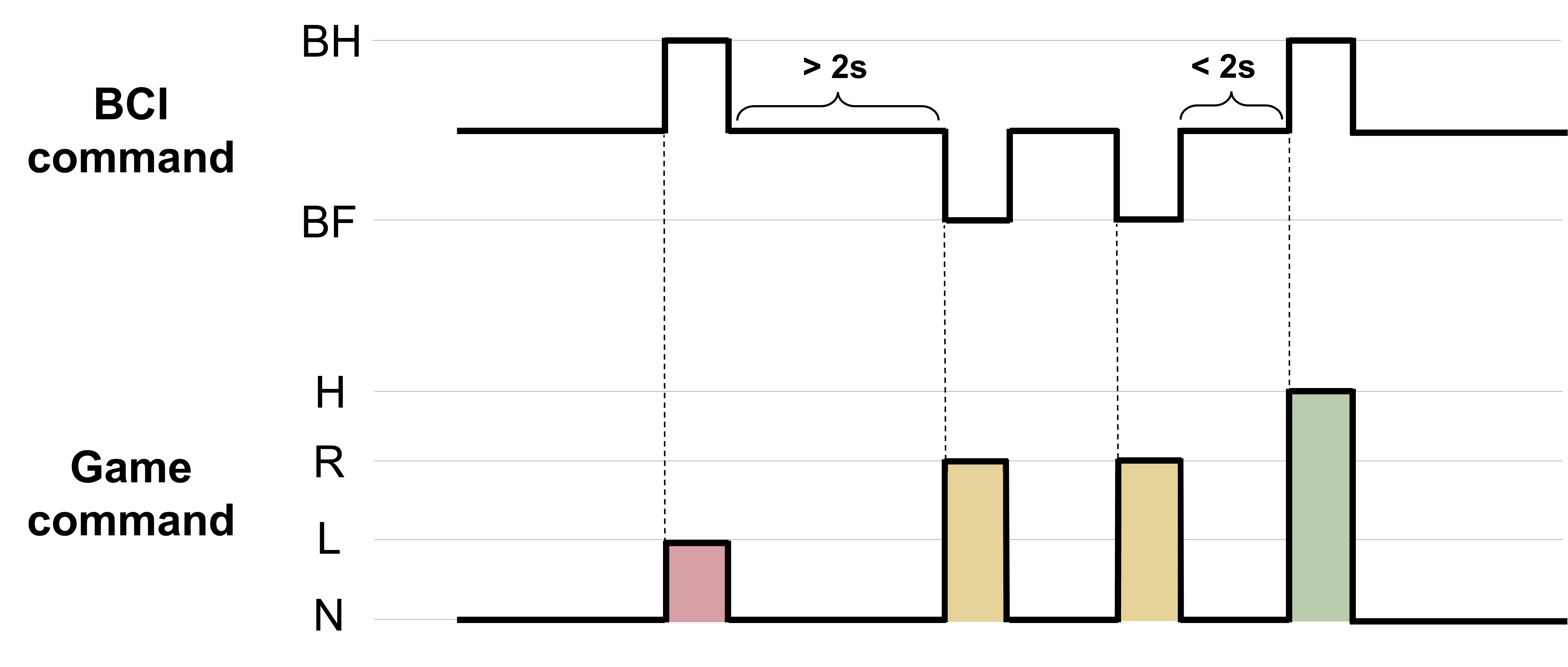

Supplement: Supplementary file 1 — Additional file 1. Schematic representation depicting the design of the implemented game control paradigm. Both hands (BH) and both feet (BF) BCI commands were associated with the delivery of “left” (L) and “right” (R) game commands, respectively. The third active command, the “headlight” (H) was instead obtained through a sequential combination approach, by sending two different BCI commands within a configurable time window (e.g., 2 seconds). While no BCI commands are generated, the pilot is considered resting and the game receives the “noinput” (N) command. [file 12984_2022_1047_MOESM1_ESM.png]

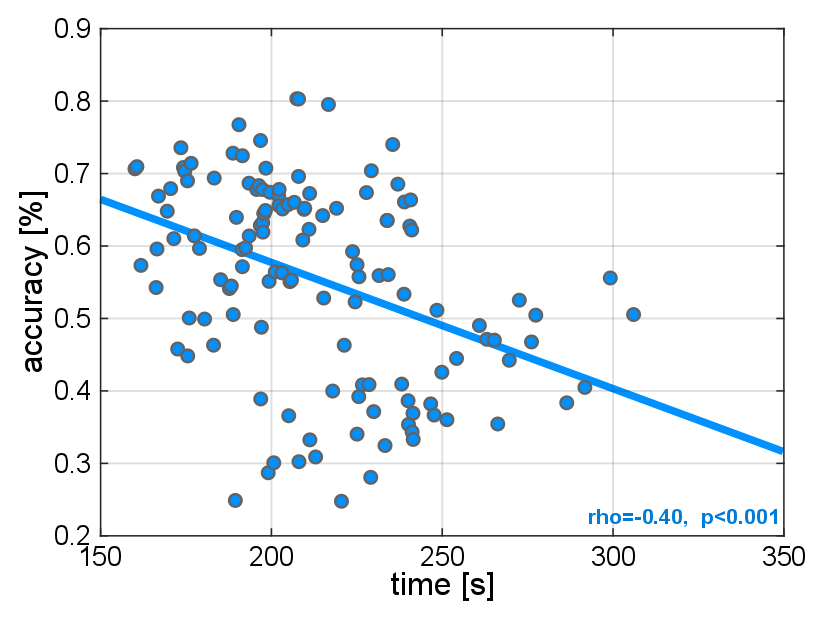

Supplement: Supplementary file 2 — Additional file 2. Relationship between our pilot’s race completion times and the classification accuracy of the decoder. Higher decoding performance generally corresponds to a shorter racing time, as shown by the linear fit and the Pearson correlation coefficient (significance tested with Student t test distribution). [file 12984_2022_1047_MOESM2_ESM.png]

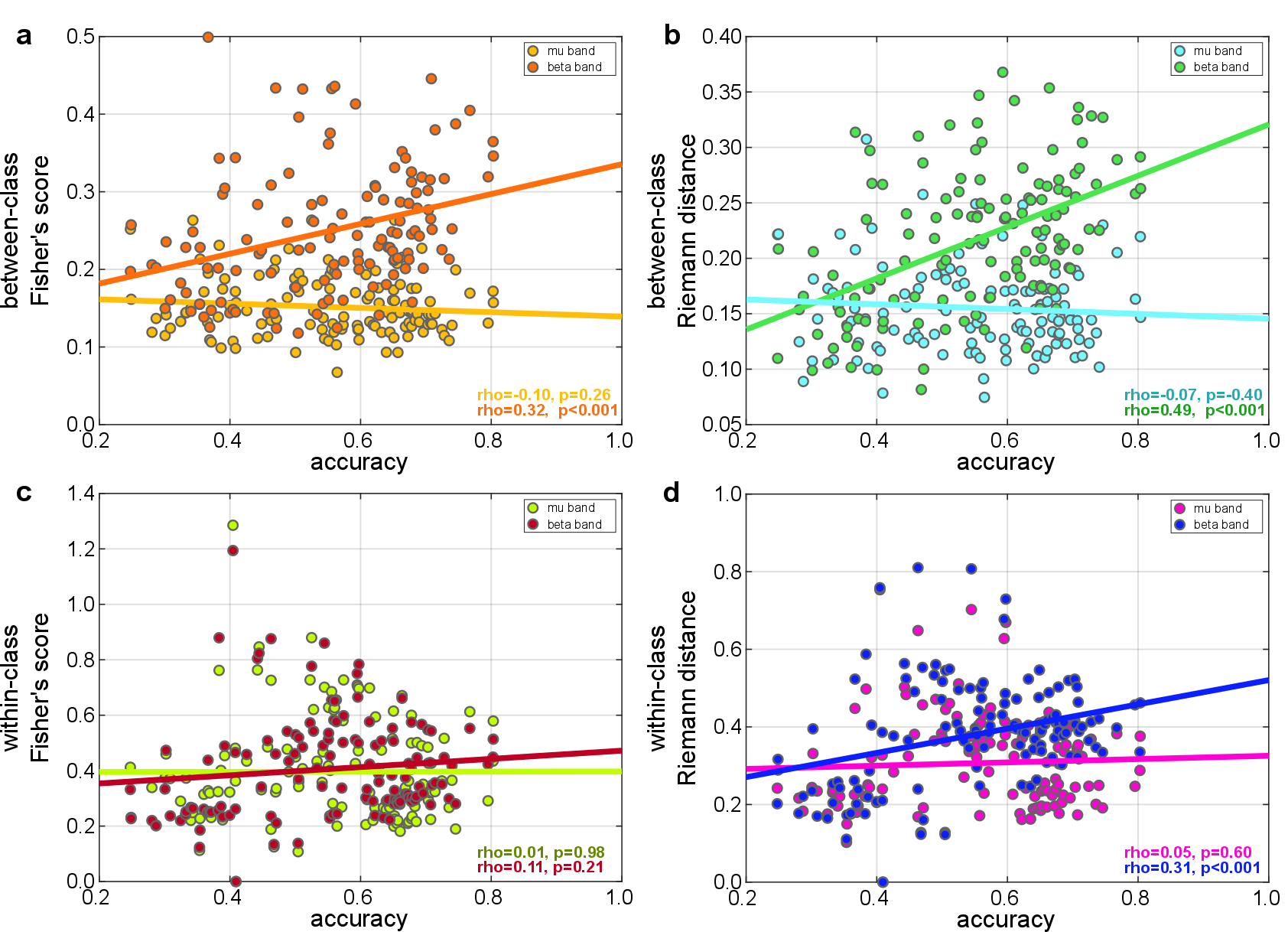

Supplement: Supplementary file 3 — Additional file 3. Relationship between the classification accuracy of the decoder and the between-class distance a, b and within-class distance c, d of our pilot’s EEG features. Linear fit and the Pearson correlation coefficients (significance tested with Student t test distribution) show a positive correlation between accuracy and band between-class distances in both the channels’ domain (a) and Riemann domain (b). A positive correlation between accuracy and the band within-class distance was found only in the Riemann domain (d), but not in the channels’ domain (b). [file 12984_2022_1047_MOESM3_ESM.png]

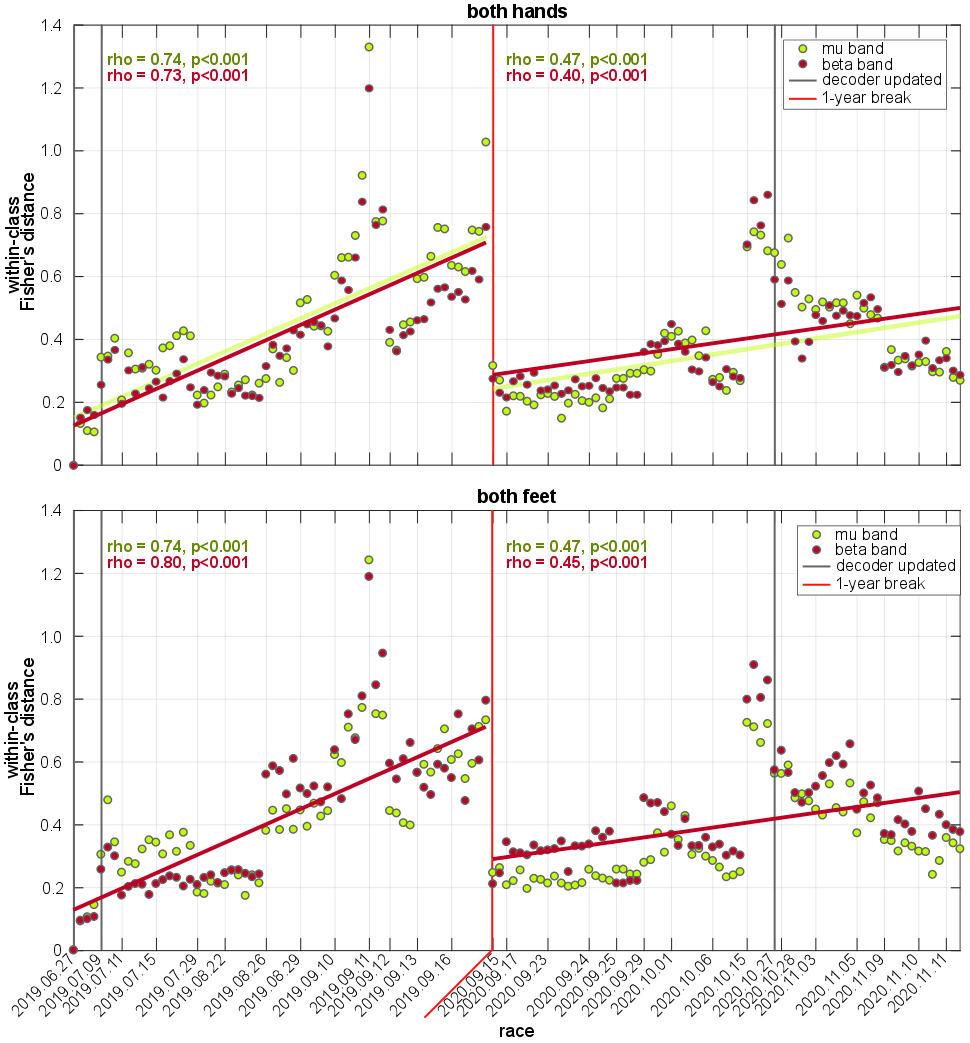

Supplement: Supplementary file 4 — Additional file 4. Evolution over races of the within-class distance in channels’ domain for the both hands class (top) and for the both feet class (bottom) in the μ-band (8–12 Hz) and β-band (16–26 Hz). Their corresponding linear fits and Pearson correlation coefficients (significance tested with Student t test distribution) were evaluated for the two years (2019, 2020) separately. Vertical thin lines indicate the date of each racing session, while vertical thick black lines represent the dates of decoder update. The break of 1 year is marked by a vertical red line. [file 12984_2022_1047_MOESM4_ESM.png]

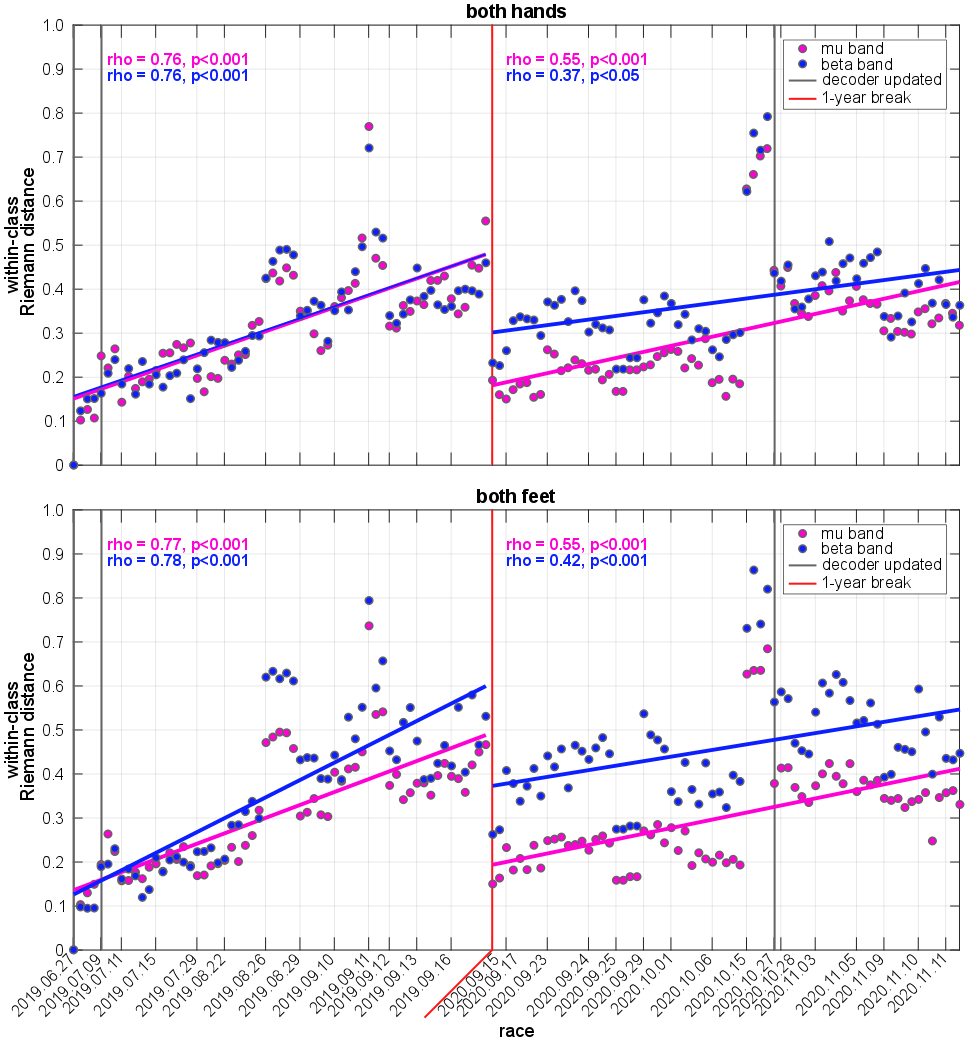

Supplement: Supplementary file 5 — Additional file 5. Evolution over races of the within-class distance in Riemann domain for the both hands class (top) and for the both feet class (bottom) in the μ-band (8–12 Hz) and β-band (16–26 Hz). Their corresponding linear fits and Pearson correlation coefficients (significance tested with Student t test distribution) were evaluated for the two years (2019, 2020) separately. Vertical thin lines indicate the date of each racing session, while vertical thick black lines represent the dates of decoder update. The break of 1 year is marked by a vertical red line. [file 12984_2022_1047_MOESM5_ESM.png]
